# Anti-Herpes Zoster Vaccination of Fragile Patients in Hospital Setting: A Nudge Intervention in Italy

**DOI:** 10.3390/vaccines12040442

**Published:** 2024-04-19

**Authors:** Francesco De Caro, Francesca Malatesta, Nadia Pecoraro, Mario Capunzo, Luna Carpinelli, Simona Caruccio, Giuseppina Cersosimo, Maria Costantino, Claudio Giordano, Walter Longanella, Vincenzo Patella, Arcangelo Saggese Tozzi, Giulia Savarese, Pio Sinopoli, Emilia Anna Vozzella, Giuseppina Moccia

**Affiliations:** 1 Public Health Laboratory for the Analysis of Community Health Needs, Department of Medicine and Surgery, University of Salerno, Baronissi Campus, 84081 Baronissi, Italy; fdecaro@unisa.it (F.D.C.); fmalatesta@unisa.it (F.M.); gmoccia@unisa.it (G.M.); 2 Department of Medicine and Surgery, University of Salerno, Baronissi Campus, 84081 Baronissi, Italy; mcapunzo@unisa.it (M.C.); lcarpinelli@unisa.it (L.C.); scaruccio@unisa.it (S.C.); clgiordano@unisa.it (C.G.); gsavarese@unisa.it (G.S.); psinopoli@unisa.it (P.S.); 3 Hospital “San Giovanni di Dio e Ruggi d’Aragona”, 84081 Salerno, Italy; mcostantino@unisa.it (M.C.); walter.longanella@sangiovannieruggi.it (W.L.); direzione.sanitaria@sangiovannieruggi.it (E.A.V.); 4 Department of Political and Sociale Studies, University of Salerno, 84084 Fisciano, Italy; gcersosi@unisa.it; 5 ASL Salerno, 84091 Salerno, Italy; v.patella@aslsalerno.it (V.P.); a.saggesetozzi@aslsalerno.it (A.S.T.)

**Keywords:** Herpes Zoster, Shingrix, vaccine, nudge intervention, prevention

## Abstract

Background: A nudge intervention against Herpes Zoster, created and implemented in Italy, is presented in order to administer the Shingrix vaccine on a sample of frail patients, as required by the National Prevention Plan. Individual and contextual factors associated with vaccine adherence were investigated. Method: 300 frail adult subjects underwent a full vaccine cycle with recombinant-Shingrix vaccine (RZV vaccine). Hospital Presidia of the Salerno University Hospital Authority, a Hospital Presidium of the Salerno Local Health Authority, and the Public Health Laboratory of the University of Salerno (Campania) participated in the intervention. An ad hoc questionnaire was administered with the following scales: EQ-5D, PSS-10, MSPSS, and representations of HZ and its consequences. Results: Some variables, such as peer support, doctor–patient relationship, level of education, and perception of health, are important in vaccine adherence and information processing. The following factors emerged from the factor analysis: Trust in collective knowledge and collective responsibility (F1); beliefs about virus risk and vaccine function (F2); information about virus and symptomatology (F3); and vaccine distrust (F4). Factor 4 correlates negatively with social support indices (R = −0.363; *p* < 0.001). There is a significant relationship between factor 3 and satisfaction with national information campaigns (F = 3.376; gdl = 5; *p*-value = 0.006). Conclusions: Future vaccination campaigns should be built with the aim of personalizing information and developing contextualized strategies, starting from understanding the stakeholders involved, cultural contexts, and organizational settings.

## 1. Introduction

Herpes Zoster (HZ) is a viral disease that occurs due to the reactivation of varicella-zoster virus (VZV) [1,2]. It can be contracted at any age; however, the incidence and the risk of complications, including mortality, increase rapidly after the age of 50 [3,4]. HZ has a more significant and lasting impact on frail patients with prior chronic diseases [3,5]. Supporting the implementation of measures to reduce the burden of vaccine-preventable infectious diseases, Italy has endorsed the National Vaccine Prevention Plan (PNPV) for 2023–2025, approved in the State-Regions Conference on 2 August 2023 and recognizes vaccination as a public health priority. The Ministry of Health introduced the new vaccine against HZ, named Shingrix (recombinant-Zoster vaccine), which is actively offered to individuals 65 years of age and at-risk individuals 50 years of age and older in the presence of frailty and prior chronic diseases. Shingrix has dramatically changed the HZ story, as it is also indicated for immunocompromised individuals older than 18 years at increased risk of HZ, unlike the previous Zostavax (a live, attenuated varicella-Zoster vaccine) [6,7].

The nudge approach can be effective in promoting vaccine adherence and reducing vaccine hesitancy, as advocated by the World Health Organization (WHO) in 2019 health care [8,9,10,11,12]. It involves the use of gentle “nudges”, which influence the architecture of choice while respecting freedom of choice [13,14,15,16,17,18,19,20]. The nudge interventions can be carried out through various modalities, such as reminders and recall, different ways of accessing information, message delivery procedures, and the use of emotional associations (e.g., videos and images) or even economic incentives [15]. In the vaccination field, various nudge interventions have been carried out worldwide, some of which have focused on the vaccination-promoting function produced by the form and/or content of the messages sent to patients. The content of the messages intersects with the heuristics and beliefs that underlie vaccine trust [15,21,22]. The form of the message (SMS, video, motivational cards, or other) is important as it activates different thought processes, such as Type 1 “automatic” or Type 2 “reflective” nudges [14,15,16,17,18,19,20]. Type 1 nudges aim to influence automatic behavior and utilize behaviors that are not conscious and deliberate.

Type 2 nudges are capable of activating both automatic response systems and reflective thinking that subsequently shapes behavior [23,24,25,26,27,28,29,30,31,32]. It can create persistent behavioral change using psychological mechanisms such as memory of past utility, self-perception, and repetition. For example, doctors could ask their frail patients to promise to get vaccinated against the flu. This commitment nudge could initially support vaccination adherence, but then, via the paths to persistence, become a new habit of the frail patients, even if the initial promise has been forgotten.

According to critics, nudge interventions focus too much on individual variables [33,34] and little on the social, relational, and ethical dimensions of the approach to the setting [35,36].

In recent years, the Public Health Laboratory of the University of Salerno (Campania), in collaboration with Local Health Authorities (ASL Salerno), has implemented nudge interventions, highlighting the strategic role of individual and contextual variables associated with vaccine adherence [9,10,37,38,39]. The literature points out that individual and social variables are determined in the implementation of a nudge intervention, particularly in the context of HZ vaccination [40], as they affect vaccine adherence or hesitation [9,10].

In general, the following variables are important indicators in anti-HZ vaccine adherence: age over 50 years, level of health literacy, female gender, higher income and education level, location in specific geographic areas, pre-existing conditions, health perception, and ability to correctly perceive disease risk [9,32,37,41]. Individual determinants include cognitions, beliefs, and thought processes within which vaccine adherence behaviors are constructed, as well as prior knowledge, experiences, and beliefs [38,42] and also the perception of one’s own health status and the perception of being able to respond to stressful life events [37,38]. The model by Betsch et al. [43], called the “5C Scale”, measured the psychological antecedents that influence vaccination behavior, identifying five key dimensions: confidence, complacency, constraints, calculation, and, finally, collective responsibility [44].

The main factors behind the reluctance to receive the vaccine appear to be a low perception of disease risk, fear related to vaccine safety, lack of confidence in vaccine efficacy, and not being aware of the vaccine’s existence and availability [45,46].

Regarding contextual variables, vaccination adherence can also be influenced by vaccination campaigns and the information sources chosen by individuals [21,39,42,47]. Therefore, when communication is inadequate, it can negatively influence vaccine adherence and contribute to vaccine hesitancy [32]. Communication can become counterproductive if it is not adapted to literacy levels, even considering the growing skepticism perpetuated by the spread of misinformation through social media.

However, the campaign communicating the social benefits of the Shingrix vaccine increased vaccination intentions against Herpes Zoster infection disease. Although this result is positive, the practical relevance may be limited. Further research into the effect of social nudges to motivate vaccination is required, particularly concerning the recent post-pandemic context and varying levels of vaccine hesitancy [48,49].

Another variable concerns belonging to social networks: psychosocial theories assert that one’s network of contacts can influence individuals’ attitudes and opinions regarding a specific topic; for example, a tendency within one’s own group to negatively consider and discuss the effects of vaccines can influence and alter a person’s behavior [50,51,52].

The aim of this research is to explore factors influencing acceptance or rejection of the recombinant-Shingrix Zoster vaccine (RZV) starting from these last considerations, with a focus on individual and contextual elements. The goal of this paper is to develop future public health strategies for tailored vaccine programs based on context analysis and specific target audiences.

## 2. Materials and Methods

### 2.1. Setting Procedure

A public health intervention was implemented within a national and local vaccine program against HZ, initiated in Italy between December 2022 and December 2023, with the scope of developing and implementing a specific vaccine strategy aimed at frail patients present in the hospital companies involved. The vaccine strategy implemented is based on a Type 2 nudge intervention.

The nudge intervention was constructed by borrowing Santinello’s [53] ecological matrix model [54] designed in the social and community sciences, which involves five levels of analysis/intervention.

The intervention cascaded to additional levels beginning with national and local policies (previously mentioned) constituting the macrosystem and community level, such as organizational, micro-system, and individual, in order to achieve the goal of vaccination.

At the community level, an application protocol was developed based on ministerial recommendations. This protocol involved collaboration among the Hospital Presidia of the Salerno University Hospital Authority, a Hospital Presidium of the Salerno Local Health Authority, and the Public Health Laboratory of the University of Salerno (Campania, Italy).

From an organizational point of view, the intervention included a series of actions:

(a) Involvement of the specific operating units to identify frail patients to whom the vaccination campaign was targeted;
(b) Training and information of health personnel in order to plan the operational strategy;
(c) Planning and implementation of a traveling vaccination within the hospital setting that would directly reach the patient during the first visit or follow-up. This vaccine involves the administration of two doses (the second dose can be administered between 2 and 6 months after the first dose);
(d) Patient involvement with specific vaccination information;
(e) Vaccination and administration of a questionnaire.

The innovation made in the intervention concerns the way in which the vaccination of frail patients took place. The vaccination center, in fact, consisted of a mobile vaccination unit, equipped with a portable refrigerator so as not to interrupt the cold chain, which followed the patient’s care pathway in the referral ward, modifying/facilitating access to the service.

At the micro-systemic and individual level, the intervention carried out focused on the doctor–patient relationship and communication within a physical and caring context already known to frail patients, as well as on knowledge of the characteristics of the patients to whom the interventions are targeted.

### 2.2. Participants

At the vaccination centers, 300 frail adult subjects undergoing anti-HZ vaccine courses with recombinant-Shingrix Zoster vaccine (RZV vaccine) were kept under observation.

### 2.3. Tools

Frail patients were administered a questionnaire comprised of the following sections:

(1) Individual data, including demographic information, education level, presence of pathologies, previous other vaccinations, sources of information about HZ vaccines, public sentiments that influence vaccination behavior and health information levels of state, informed consent form and privacy policy, vaccine information (injection site, lot number, expiration date, route of administration, and health professional data). Enrolled subjects, when taking their first vaccine dose, were asked to anonymously fill out a purpose-built questionnaire and enter it on the Google Forms platform via a QRCode. All participants were made aware, through informed consent, that participation was voluntary and anonymous, that they could withdraw their consent at any time as well as of the use of data in anonymous and aggregate form.
(2) Standardized tests: EuroQol-5D (EQ-5D); Perceived Stress Scale 10 (PSS-10); Multidimensional Scale of Perceived Social Support (MSPSS); and finally, a scale created ad hoc to detect perceptions, information, and beliefs about the virus and vaccine:
  - EQ-5D (EuroQol-5D) [55] is a standardized questionnaire used to measure health-related quality of life (HRQL). The instrument is divided into two sections; the first section (5 items) involves a subjective assessment of five dimensions: mobility, self-care, activities of daily living, pain/concern, and anxiety/depression. Each item involves responses from 1 to 3 (1 = no problem; 3 = extreme limitation). The second section includes an assessment by visual analog (VAS) graphically represented by a graded scale ranging from 0 to 100, on which the subject must indicate his or her perceived level of health (0 = worst possible health status; 100 = best possible health status);
  - PSS-10 (Perceived Stress Scale 10) [56] assesses individual perception with respect to particular everyday life situations and reactions in response to these stressful events. The respondent is asked to indicate how often he or she has felt or thought a certain way during the previous 4 weeks. The questionnaire consists of 10 items and involves responses rated on a 5-point Likert scale according to severity. Individual scores on the PSS can range from 0 to 40, with higher scores indicating a greater perception of stress;
  - MSPSS (Multidimensional Scale of Perceived Social Support) [57] is an instrument traditionally used to detect social support as a multidimensional construct. It consists of 12 items grouped into three factors: family, friends, and significant others. The respondent is asked to express his or her level of agreement on a 7-point Likert scale (1 = completely disagree; 7 = completely agree). The instrument allows for both a total score indicating the subjective assessment of the adequacy of perceived social support and a relative score for each subscale, ranging from 1 to 7, with higher scores indicating greater perceived social support. Previous studies have indicated that the MSPSS is a valid and reliable instrument for measuring the perception of social support in people with chronic illness [58];
  - A scale was created ad hoc to survey perceptions, information, and beliefs about the virus and vaccine. The scale consists of 16 items, with each item predicting responses from 1 to 4 (4 = totally agree; 1 = totally disagree). The scale has not been previously validated.

### 2.4. Statistical Analysis

Data analysis was performed using IBM SPSS v.28 software (IBM®SPSS®, Bologna, Italy).

The data related to the first section of the questionnaire, concerning socio-demographic variables and public sentiments that influence vaccination behavior and health information levels of state, underwent descriptive statistical analysis: analysis of absolute and percentage frequencies and mean and standard deviation for the age variable. Chi-square analysis was conducted to cross-reference these variables (only results with a *p*-value < 0.005 were considered).

Regarding the scales present in the second section: EQ-5D (EuroQol-5D), PSS-10, MSPSS, in addition to calculating scores and conducting descriptive analysis for the individual scales and subscales, inferential analyses such as Pearson Correlation (R-Pearson) and Analysis of Variance were performed to relate the scores obtained on the scales. The scores of the EQ-5D subscales were related to age and present illnesses. Again, results with a *p*-value < 0.005 were considered.

For the final section, Exploratory Factor Analysis (EFA) was conducted on the 16-item questionnaire regarding perception, information, and beliefs about the virus and the vaccine. The least squares method with Varimax rotation was utilized. Items with a commonality > 0.4 were included, leading to the elimination of four items from the analyses. Kaiser–Meyer–Olkin measures indicated an adequate sampling value (0.813), while Bartlett’s test showed a significance of <0.001. Four factors explaining 59.6% of the variance were extracted. The factor structure was then confirmed through Confirmatory Factor Analysis (CFA) on 12 items. Four factors were extracted. These four factors were then correlated (R-Pearson) both with the scores obtained on the scales for perceived psychological well-being and with the relationships with the variables from the first section through Analysis of Variance.

## 3. Results

### 3.1. Sample Description

Patients had a mean age of 54.9 years (SD = 13.8). Women accounted for 37.5% of the sample, and men for 62.5%. Women had a mean age of 51.6 years (SD = 16.13) and men 56.8 years (SD = 11.84).

Table 1 presents demographic characteristics. Regarding marital status, 67.9% of the participants were married, 6.5% were separated/divorced, 19.6% were single, 3% were widowed, and 3% were cohabiting. Among the participants, 89.9% were cohabiting with family or roommates, while 10.1% lived alone. The level of education was as follows: 78% completed education up to the second grade, while the remaining 22% have at least college-level or higher (post-graduate training). As for occupation, 14.3% are office workers, 4.2% are teachers, 11.9% are homemakers, 11.9% perform laborer/craftsman work, 10.7% are self-employed, 2.4% are students, 28.6% are retired, 8.3% are unemployed, and 7.7% state “other”.

Regarding contact with the HZ, 24.4% of the participants had HZ, while 75.6% did not. With respect to the vaccine, 98.0% were on the first dose, and 2.0% were on the second dose. Regarding the adherence of these patients to other vaccination campaigns, it was found that out of the total number of responses, 98.2% have taken the COVID-19 Vaccine, 50.6% have taken the flu vaccine, and, finally, 3.0% have also taken other vaccines.

Table 2 displays past medical conditions. Among all responses, 19.7% reported chronic heart disease, 6.8% hypertension, 20.1% diabetes mellitus, 7.9% pulmonary diseases, 77.4% were following immunosuppressive therapy, 6.1% had rheumatological diseases, 6.1% had oncological diseases, 1.8% were undergoing dialysis treatment, 2.4% were awaiting organ transplantation, and 4.3% underwent hematopoietic stem cell transplantation.

#### Public Sentiments That Influence Vaccination Behavior and Health Information Levels of State

Regarding attitudes toward the virus and toward vaccination, those who have not had HZ say they are more in favor of vaccination (Chi-square = 8.6; df = 3; *p*-value = 0.045). Men show less fear of the effects of HZ (Chi-square = 9.35; df = 3; *p*-value = 0.025), as do those who have not had the flu vaccine (X = 7.9; df = 3; *p*-value = 0.048).

Regarding the sources of information used about vaccines and vaccination, the major sources were media (TV-Radio) 10.1%, web and social web 14.3%, medical personnel 73.8%, and, finally, “word of mouth” 1.8% (Table 3).

Source afference differs by gender (Chi-square = 10.32; df = 3; *p*-value = 0.017). Men use doctors’ information more, while women are afferent to different sources with more attention to media and the social web compared to men. Those with a “bachelor’s/postgraduate” level of education are less trusting of information received from the media (Chi-square = 9.49; df = 3; *p*-value = 0.023).

In relation to the level of satisfaction with the vaccination campaign, 9.1% considered themselves totally dissatisfied, 14.0% somewhat dissatisfied, 14.0% neither satisfied nor dissatisfied, 27.4% somewhat satisfied, 32.3% satisfied, and, finally, 3.0% had no experience with it (Table 3).

The degree of satisfaction is significant with respect to the most frequently used sources of information (Chi-square = 39.23; df = 15; *p*-value < 0.01). Those who are most satisfied with the national campaigns use the web and social web as sources; those who are most dissatisfied use radio and TV as information media. Those who acquire information from medical personnel are also fairly satisfied.

### 3.2. Perceived Psychological Well-Being

Among individual variables, we consider the following: perceived quality of life assessment, perceived stress, and perceived social support.

Quality of life, as measured through the EuroQol-5D (EQ-5D), can be expressed through two EQ5 indices:

(a) Perception of general health status (VAS): a mean score of 71.96 (SD = 17.57), a score that is between the 25th and 50th percentile, as well as the EQ5-D index. The EQ5-D score is a summary of the five areas and is 0.79 (SD = 0.24).

These indices correlated negatively with age: VAS (R = −0.328; *p* value = 0) and EQ5-D (R = −0.219; *p* value = 0.004).

Regarding the diseases present, those with chronic heart disease (excluding hypertension) have significantly lower mean at the two indices, VAS (F = 6.24; DF = 1; *p*-value = 0.012) and EQ5-D (F = 4.33; DF = 1; *p*-value = 0.039), as well as those with chronic lung diseases VAS (F = 9.24; DF = 1; *p*-value = 0.003) and EQ5-D (F = 14.608; DF = 1; *p*-value = 0.00).

A significantly low score on the health status perception scale is found in those with hypertension: VAS (F = 4.73; DF = 1; *p*-value = 0.031), as well as for diabetes mellitus VAS (F = 9.44; DF = 1; *p*-value = 0.022). Those on dialysis treatment have a significantly low score on the EQ5-D (F = 4.76; DF = 1; *p*-value = 0.30).

(b) Perceived Stress Scale (PSS10): Patients respond with a mean score of 13.87 (SD = 5.96). This score is in a border zone between low and medium perceived stress.

Stress index correlates negatively with perceived health, i.e., the less people perceive stress, the more they have a better EQ5-D health index (R = −0.300; *p*-value = 0) and VAS (R = −0.273; *p*-value = 0).

(c) Perceived Social Support Scale (MSPSS): People are in the high social support range (mean = 65.37; SD = 16.19). Within the subscales, the score with the highest mean is defined by perceived family social support (Table 4).

The social support index correlates negatively with the stress index (R = −0.287; *p*-value = 0).

### 3.3. Perception, Information, and Beliefs about the Virus and Vaccine

Four factors were extracted as follows (Table 5 and Table 6):

Factor 1: confidence in collective knowledge and collective responsibility;
Factor 2: beliefs about virus risk and vaccine function (social norm);
Factor 3: information about the virus and symptomatology;
Factor 4: vaccine distrust.

As shown in Table 6, the factors were correlated with the indices of well-being status (EQ-5D), perceived stress (PSS10), and perceived social support (MSPSS).

The results show that trust in collective knowledge and collective responsibility (F1) correlates positively with perceived health and perceived peer social support, while it correlates negatively with age.

People who feel good and have a good perception of social support trust others and are protective of others, a positive relational circle. The older one is, the less protective one is of others or trusts collective knowledge.

Beliefs about the risk brought by contracting the virus and the positive function of the vaccine (F2) correlate positively with age and perceived well-being, i.e., older people are more focused on perceived risk, just as those with a generally low perception of health are alert to the risks they may face.

Mistrust of the vaccine (F4) correlates negatively with all indices of social support. That is, people who perceive themselves as less supported are more distrustful of the vaccine.

Performing an ANOVA between factors and some questions in the first section shows that distrust of the vaccine is higher in those who have already had shingles (mean = 0.3054222; SD = 1.0) than in those who have not (mean = 0.0986; SD = 0.8878) (F = 5.852; gdl = 2; *p*-value = 0.017) (F = 5.852; gdl = 2; *p*-value = 0.017).

There is a significant relationship between the factor Information about the virus and its symptomatology (F3) and satisfaction with national information campaigns (F = 3.376; gdl = 5; *p*-value = 0.006), particularly with a higher level of satisfaction.

## 4. Discussion

The ecological model [53,54], when applied to study and prevention interventions in public health, demonstrates the intricate nature of constructing health actions. It emphasizes the importance of understanding the interplay between individual and contextual factors, including socio-cultural and organizational aspects.

In our nudge intervention, carried out on fragile patients in hospital, we aimed to detect individual and contextual factors that can influence the success of a vaccination campaign.

The main factor for vaccination adherence [44], as observed in the questionnaires, is represented by the relationship with healthcare personnel.

The vaccination strategy implemented has emphasized the relationship with the healthcare personnel who already assist the patient in his pathology and frail condition. In this regard, we underline how the intervention involved planning and implementing an “itinerant vaccination” that facilitated the establishment of a network comprising physicians and nursing staff. This network contributed to building trust, increasing knowledge, and fostering motivation in frail patients. The referral department, within the intervention, became a symbolic tool for safeguarding and incentivizing vaccine adherence among frail patients. Suppliers have been entrusted with direct responsibility for planned activities to allow this, enhancing communication strategies in patient enrollment and identifying critical issues that were addressed before implementing the vaccine model.

The relational sphere, a nodal point of the micro-systemic level, appears crucial for the observed frail patients. Individuals who perceive greater support, especially from peers, exhibit good health, a low-stress perception, trust in collective knowledge, and demonstrate responsibility toward protecting others [21,58]. Overall, social support from various sources, including physicians, family members, and peers, emerges as an excellent protective factor that can be targeted for interventions. It could be useful to carry out interventions that involve family members to support patients and serve as a bridge with physicians or again on the peer group with informative group counseling.

Furthermore, analyzing the observed individual and contextual variables reveals that individuals with a higher average age appear to be at an increased risk, marked by elevated stress levels and lower perceived social support [58]. This demographic tends to be less informed and less concerned about community welfare, scientific knowledge, and collective knowledge. Instead, they tend to rely on an entrenched belief system associated with the conception of risk and their own health [45,51].

Examining the type of information message on Herpes Zoster, a crucial contextual variable in the nudge’s intervention, frail patients express higher satisfaction with vaccination campaigns promoted through the web, social web, and physicians, as discussed earlier. Conversely, they report lower satisfaction with campaigns through radio and TV. Previous research, on the other hand, had highlighted that patients were poorly informed by medical staff [59]. The individual variable of gender significantly influences access to and trust in information, with women relying more on information from the web/social web and media. In terms of education level, it actively contributes to trusting information sources, with a preference for communications provided by physicians over radio and TV. Additionally, individuals with higher education levels exhibit less trust and satisfaction in national vaccine campaigns.

This informational process seems interesting in that it probably activates more Type 2 processing, which requires in-depth information, such as searching information portals, alongside automatic processes. Nudge, in our opinion, should not only act on the end goal (vaccinate) but precisely on the modification of cognition [60], transforming the attitude of willingness to vaccinate into actual vaccination adherence [61]. This represents an active attitude towards knowledge, avoiding reliance on informational sources, often found on the web, that amplify vaccination fears [48,59,62].

Regarding contact with the virus and vaccination history, individuals who have never had the disease tend to be more supportive of the vaccine. Conversely, those who have not received other vaccines previously express less fear of the effects of HZ, indicating a lower focus on the perceived risk and effects of the disease.

Delving into individual variables, the perception of health stands out as generally low and correlated with higher stress levels. Particularly, patients with diabetes mellitus, chronic heart disease, chronic lung disease, and those undergoing dialysis treatment show a strong correlation with a low perception of quality of life. We hypothesize that this could be a predisposing factor for vaccine adherence, as patients who are already ill may be apprehensive about the risks of an additional disease like HZ and its potential impact on quality of life. In this regard, recent studies have shown that patients with chronic diseases are more compliant towards vaccination [59]. It seems to us to infer that vaccine adherence highlighted, once again, the role of certain psychological antecedents that come to change in contact with contexts and through the relationships that develop in them, as in the case of the hospital setting. Therefore, it is crucial to comprehend the systems of meaning [63] within which individuals construct knowledge, beliefs, languages, motivations, and behaviors.

This nudge intervention reveals strengths for vaccine adherence [43,44], such as the physical availability of the vaccine, a heightened perception of risk—especially among older individuals in this sample—a sense of collective responsibility [21] prevalent in younger individuals and those perceiving better health conditions and strong social relationships. Other variables from the 5C model are also present, including the degree of commitment to seeking information (less strenuous as provided on an ad hoc basis by physicians) and confidence in the vaccine and the treating health system. These factors are fundamental for patients who have relied on the hospital setting for an extended period.

Considering physician–patient communication as a critical junction between vaccine adherence policies and actual adherence, it is essential to reflect on the role of local cultures. These cultures shape users’ cognitions, emotions, and personal motivations, influencing how physicians interact with patients. This understanding is significant for activating strategic and effective information campaigns [21,50,64], as well as nudge interventions. For instance, when dealing with frail patients with a higher level of education, there is a need to present information at multiple levels to facilitate decision-making. Conversely, when engaging with older individuals, information should be tailored to personal risks and improvements in quality of life [45,46].

It is evident that the key components of the proposed model are vaccine accessibility, the establishment of safe pathways tailored to the patient type, the implementation of a communication strategy tailored to the vaccine type, the training and information of health personnel [65], and the contextualization of interventions concerning the environment, including the physical setting.

One limitation of this intervention lies in administering the questionnaire exclusively to frail patients who have adhered to vaccination. The sample characteristics also pose a limitation. For example, in the case of neoplastic patients, anti-HZ vaccination could not be proposed and implemented, as oncology physicians believe specific guidelines are necessary to indicate when and how vaccination should be carried out in relation to the patient’s cancer therapy.

Expanding the research to include a sample of nonadherent frail patients and comparing the results or assessing subsequent anti-HZ vaccination campaigns would be beneficial. Furthermore, it would be interesting to expand the research to include family members or caregivers of frail patients in order to understand their perceptions of HZ and vaccination, as well as the communicative methods they use to encourage their family members to get vaccinated.

From a methodological standpoint, the vaccination strategy utilized for HZ aligns with current recommendations from scientific societies, advocating for the integration of vaccination activities within the hospital setting.

Future developments for patients with chronic diseases, identified as being at risk of infection and severe forms of infectious diseases, involve implementing PDTCPs (Preventive Diagnostic Therapeutic Care Pathways). It proposes anticipating the implementation of PDTCPs (Preventive Diagnostic Therapeutic Care Pathway), facilitating any organizational form that initiates the vaccination pathway for the patient at different stages, such as proposing the recommended vaccine offer during hospitalization or outpatient follow-up. This approach reduces time constraints and enhances compliance.

A future line of research could investigate whether the implementation of PDTCPs can have an effect on increasing vaccination adherence both in patients and caregivers.

## 5. Conclusions

In conclusion, knowledge of individual and contextual determinants is essential to understanding health-related behaviors. These components serve as the foundational matrix, adaptable and customizable for each unique context and target audience, to build an effective and functional vaccine strategy that fosters adherence.

This study suggests the need to implement tailored local campaigns in addition to institutional ones, utilizing both traditional and digital channels. These local campaigns should be meticulously formulated through an analysis of the specific context, customizing information based on the target audience, and identifying diverse strategies in consideration of various contexts (environmental, physical, organizational, human resources, etc.) in which vaccination occurs involving all stakeholders who are part of the vaccination process.

## Figures and Tables

**Table 1:** Socio-demographic and Herpes Zoster experience of total sample.

Main Categories	Variables		%
Socio-demographic	Gender	Men	62.5%
		Women	37.5%
	Work	Employee	14.3%
		Teacher	4.2%
		Housewife	11.9%
		Worker/Craftsman	11.9%
		Self-employed	10.7%
		Student	2.4%
		Retiree	28.6%
		Unemployed	8.3%
		Other	7.7%
	Marital status	Married	67.9%
		Separated/Divorced	6.5%
		Single	19.6%
		Widowed	3.0%
		Cohabiting	3.0%
	Live	Cohabiting with family or roommates	89.9%
		Live alone	10.1%
	Level of schooling	Secondary school degree	78.0%
		University degree/Post-graduate training	22.0%
Vaccinations and Hz infection	HZ vaccination	First	98.0%
		Second	2.0%
	HZ infection	Yes	24.4%
		no	75.6%

**Table 2:** Output of a frequency table of a multiple response set of previous pathologies.

Variable		Total Count%
Previous pathologies	Chronic heart disease	19.7%
	Hypertension	62.8%
	Diabetes mellitus	20.1%
	Lung diseases	7.9%
	Immunosuppressive therapy	77.4%
	Rheumatological diseases	6.1%
	Oncological pathologies	6.1%
	Dialysis treatment	1.8%
	Organ transplantation	2.4%
	Hematopoietic stem cell transplantation	4.3%

**Table 3:** Frequency of answers to items on vaccination campaign.

Item on Vaccination Campaign Information		%
Sources used in the vaccination campaign	Media (TV-Radio)	10.1%
	Web and social web	14.3%
	Doctors	73.8%
	Pass the word	1.8%
Degree of satisfaction with sources	Totally dissatisfied	9.1%
	Somewhat dissatisfied	14.0%
	Neither satisfied nor dissatisfied	14.0%
	Fairly satisfied	27.4%
	Satisfied	32.3%
	I have had no experience with this	3.0%

**Table 4:** Mean and standard deviation of MSPSS scores.

MPSS Scores Categories	Mean	SD
Family	23.14	5.453
Friends	19.81	6.737
Significant others	22.42	6.145
Total score	65.37	16.194

**Table 5:** Rotated matrix of factors (saturations less than 0.4 were omitted) and percentages of variance explained by individual factors.

Item	Factors Extracted			
	I	II	III	IV
7. I believe that by vaccinating myself, I protect those close to me.	0.596			
13. I trust the progress of science in the field of vaccines and, in particular, Herpes Zoster.	0.622			
14. I trust the information I have received from healthcare personnel.	0.816			
16. I trust the information I received from my family members.	0.613			
2. I believe that Herpes Zoster is a serious disease.		0.610		
4. I believe the probability of contracting Herpes Zoster is high if I don’t get vaccinated.		0.637		
6. I believe that the vaccine can protect me in the future.		0.493		
8. I think everyone should get the Herpes Zoster vaccine.		0.602		
1. In general, I am sufficiently informed about the Herpes Zoster virus.			0.650	
2. I am sufficiently informed about the symptoms and consequences of Herpes Zoster.			0.928	
11. I am against vaccination.				0.929
12. The fact that some vaccinations are not mandatory means that they are not necessary.				0.686
% variance explained	36.8	10.7	7.2	5.2

**Table 6:** Correlation between the scores obtained on the reported scales and the four factors.

Variables	Factors			
	I	II	III	IV
Age	−0.163 *	0.199 *	0.046	0.087
EQ-5D (EQ index)	0.094	−0.118	0.110	0.032
EQ-5D (VAS)	0.172 *	0.133 *	0.041	−0.020
PSS10 stairs	−0.080	0.108	−0.073	0.172 *
MSPSS (total score)	0.097	−0.060	0.052	−0.363 *
MSPSS (family scale)	0.091	−0.035	0.021	−0.348 *
MSPSS (friends scale)	0.141	−0.058	0.048	−0.267 *
MPSS (significant other scale)	0.020	−0.065	0.064	−0.355 *

Legend: (*) = *p* < 0.01 (one-tailed).

## Data Availability

Written informed consent was obtained from the subjects in order to publish this paper. The archived data is not public and can be requested by writing to the corresponding author.
